# *Caffeoyl-CoA 3-O-methyltransferase* gene family in jute: Genome-wide identification, evolutionary progression and transcript profiling under different quandaries

**DOI:** 10.3389/fpls.2022.1035383

**Published:** 2022-12-14

**Authors:** Saima Akhter, Asif Ahmed Sami, Tamanna Islam Toma, Bushrat Jahan, Tahmina Islam

**Affiliations:** Plant Breeding and Biotechnology Laboratory, Department of Botany, University of Dhaka, Dhaka, Bangladesh

**Keywords:** CCoAOMT gene, jute, lignin, abiotic stress, functional validation, histochemical assay

## Abstract

Jute (*Corchorus* sp.), is a versatile, naturally occurring, biodegradable material that holds the promising possibility of diminishing the extensive use of plastic bags. One of the major components of the cell wall, lignin plays both positive and negative roles in fiber fineness and quality. Although it gives mechanical strength to plants, an excess amount of it is responsible for the diminution of fiber quality. Among various gene families involved in the lignin biosynthesis, Caffeoyl-CoA 3-O-methyltransferase (*CCoAOMT*) is the most significant and has remained mostly unexplored. In this study, an extensive *in-silico* characterization of the *CCoAOMT* gene family was carried out in two jute species (*C. capsularis* L. and *C. olitoroius* L.) by analyzing their structural, functional, molecular and evolutionary characteristics. A total of 6 *CCoAOMT* gene members were identified in each of the two species using published reference genomes. These two jute species showed high syntenic conservation and the identified *CCoAOMT* genes formed four clusters in the phylogenetic tree. Histochemical assay of lignin in both jute species could shed light on the deposition pattern in stems and how it changes in response to abiotic stresses. Furthermore, expression profiling using qPCR showed considerable alteration of *CCoAOMT* transcripts under various abiotic stresses and hormonal treatment. This study will lay a base for further analysis and exploration of target candidates for overexpression of gene silencing using modern biotechnological techniques to enhance the quality of this economically important fiber crop.

## Introduction

Jute, a dicotyledonous fiber yielding plant of the genus *Corchorus*, family Malvaceae, has been one of the major fiber yielding crops worldwide. Jute is the second most important bast fiber (fiber collected from the skin of the plant) after cotton in terms of usage, worldwide consumption, and availability, accounting for 84% of global output. Jute and jute products significantly benefit the environment by slowing down ecological degradation and preserving the natural environment and atmosphere. Jute fibers are eco-friendly materials because of their biodegradable properties and are easily recyclable. The bag and cloth industry are the largest consumer of jute fibers available on the market. Jute bags have gained popularity as an eco-friendly alternative to both non-biodegradable poly bags derived from petroleum and paper bags that require significant amounts of wood. To improve the quality of jute products, knowing the components of its fiber is needed. Jute products are mainly made from the outer portion of the stem, also known as bast fiber. This is a lignocellulosic fiber composed of 61-71.5% cellulose, 13.6-20.6% hemicellulose and 12-13% lignin (Sood and Dwivedi, 2018). However, up to 12-26% lignin was also recorded in jute fibers (Selver et al., 2018). The current status of lignin in Tossa pat variety O-9897 of Bangladesh is 29.50% and 13.46% in whole stem and in fiber respectively (Shafrin et al., 2017) and in O-72 it was measured as 29% in mature jute plant with a deposition ratio 0.21% per day (Tanmoy et al., 2015).

Lignin is a complex heteropolymer of cinnamyl alcohols with high molecular weight and is a key component of the jute cell wall. Also, it is the second most prevalent biopolymer on Earth after cellulose (Baucher et al., 1998; Liu et al., 2018). Dehydrogenative polymerization of three hydroxy-cinnamyl alcohols (monolignols), namely p-coumaryl alcohol, coniferyl alcohol, and sinapyl alcohol, results in p-hydroxyphenyl (H), guaiacyl (G), and syringyl (S) units of lignin, respectively (Vanholme et al., 2010). As one of the key components of the cell wall, lignin metabolism has a variety of roles, most notably in battling numerous environmental stressors, as the plant cell wall serves as the first line of defense against external threats. Lignin has a crucial function in the structural integrity of cell walls, stem strength, water transport, and pathogen resistance (Mutuku et al., 2019). This high lignin content separates jute fiber from other non-wood bast fibers such as flax, hemp, and ramie, as well as kenaf (Del Río et al., 2009). The poor quality of jute fibers in comparison to other fiber crops is mostly owing to greater levels of lignin in its fiber (more than ramie and cotton), making it less appropriate for creating finer textiles and other value-added goods (Loumerem and Alercia, 2016). Thus, understanding the enzymes involved in the biosynthesis of lignin is a prerequisite for any type of analysis to alter the amount or components of lignin in plants.

Caffeoyl-CoA 3-O-methyltransferase (*CCoAOMT*) is one of the primary enzymes responsible for the biosynthesis of lignin in plants (Zhong et al., 1998; Meyermans et al., 2000). This enzyme is a type of S-adenosyl-L-methionine (SAM) methyltransferase that utilizes coffee acyl coenzyme A as a substrate. The enzyme is encoded by the *CCoAOMT* gene, hitherto, various techniques have been reported to lower the lignin contents by targeting the *CCoAOMT* gene in those respective plants. For instance, in *Pinus radiata*, *CCoAOMT* suppression alters lignin composition (Zhong et al., 1998; Kawaoka et al., 2006; Wagner et al., 2011). Lignin content was lowered in tobacco by suppressing two O-methyltransferase genes - caffeic acid O-methyltransferase (*COMT*) and *CCoAOMT* through antisense technology (Zhao et al., 2002; Vanholme et al., 2008; Tronchet et al., 2010). *CCoAOMT* suppression in *Nicotiana tabacum*, *Arabidopsis thaliana*, *Medicago sativa*, and *Populus* resulted in lignin reductions of 20-45% of total lignin (Meyermans et al., 2000; Zhong et al., 2000; Do et al., 2007). The *CCoAMT1* gene was downregulated in the low-lignin mutant of *Corchorus olitorius* and was found to be accountable for the mutant’s low lignin content (Choudhary et al., 2017). Furthermore, overexpression of a jute *CCoAOMT1* gene in *Arabidopsis thaliana* resulted in higher lignin content compared to the non-transgenic plants (Zhang et al., 2014). This confirms the important role of *CCoAOMT* gene in the lignin biosynthesis pathway (Zhang et al., 2014). Lignin gives mechanical support to fight against biotic stresses, it also helps plants in combating various abiotic stresses (Cabane et al., 2012).

Members of the *CCoAOMT* gene family have already been found and studied in a variety of plant species, including Arabidopsis, rice, sorghum, citrus, and poplar (Hamberger et al., 2007; Xu et al., 2009; Liu et al., 2016; Rakoczy et al., 2018). But, it is yet to be characterized fully in jute. The present study tackles this gap through an *in-silico* analysis of all the members of the *CCoAOMT* gene family from two jute species (*C. capsularis* and *C. olitorius*). Here, we report 6 *CCoAOMT* members in each of the jute species. Lignin deposition in the jute stem under normal environmental conditions and in different abiotic stresses was also shown. Overall, the functional validation and expression profiling of this gene family provides a foundation for future genetic engineering and plant breeding programs to alter lignin composition in jute plants.

## Materials and methods

### Plant growth condition and stress treatments

Among the two jute species, BJRI Tossa Pat-8 (Robi-1) variety of *C. olitorius* was chosen due to its high germination rate for expression profiling, histochemical assay and for stress responsive amino acid and phenolic measurement. Uniformly developed jute seed of the BJRI Tossa Pat-8 variety was planted in optimum greenhouse conditions for (16 h photoperiod, 28C temperature, 80% humidity). Plants were irrigated with Hogland solution on alternate days to create optimum growing conditions. Two months old plants were subjected to different abiotic stresses such as hormone (20 uM ABA), drought (20% (w/v) PEG 6000 solution) and saline (200 mM NaCl) treatment. For salinity treatment, two months old soil grown plants irrigated with 200mM NaCl in Hogland solution (Li et al., 2019). A solution of 20% Poly-Ethylene Glycol (PEG6000) in Hogland solution was used to create drought conditions. Similarly, 20 uM ABA was applied in the plant by spraying to mimic the hormonal treatment. The control plants were irrigated with hogland solution. Leaf and stem samples (three biological replicates) from control and treated plants were collected at 24-, 48- and 72-hours intervals (for expression profiling) and after 72 hours for other experiments, and instantly frozen in liquid nitrogen before being stored at -80°C. The untreated plants served as control compared to the treated plants.

### Histochemical assay of lignin and fluorescence microscopy

Stem sample from 72h stresses two months old, lignified jute (BJRI Tossa Pat-8 variety of *C. olitorius*) were collected to elucidate any difference in the deposition of lignin in different regions (epidermis, bast region, pith). Hand sections of jute stems were dyed with phloroglucinol, a dye that combines with the cinnamaldehyde end groups of lignin to produce a crimson color in the lignified region and examined under a 10× magnification light microscope (Nikon ECLIPES 50i). The presence of lignin was observed when the tissues were dyed red. A CCD (charged-couple device) camera (Nikon DS-U3 DS Camera Control Unit; Ver.1.00) was used to capture the fluorescence images. No filter was used during the microscopy.

### Identification, gene nomenclature of *CCoAOMT* gene family in two species of jute along with molecular attributes

According to Zhang et al. (2021) the sequences and all the required files of identified *CCoAOMT* genes in two species of jute (*C. capsularis* var. ‘Huangma 179’ (HM179) and *C. olitorius* var. ‘Kuanyechangguo’ (KYCG)) were retrieved from Genome Warehouse in the National Genomics Data Center. To confirm their function, the putative CCoAOMT sequences were analyzed using the Pfam database (Mistry et al., 2020). The gene names were assigned in descending order based on their chromosome position (Hasan et al., 2021). Information regarding locus ID, protein size and full-length CDS were obtained from the National Genomics Data Center. Expasy’s Protparam tool was used to acquire information on molecular weight, theoretical pI, and number of amino acids (Gasteiger et al., 2005). CELLO v.2.5: subCELlular Localisation predictor (Yu et al., 2006) and WoLF pSORT4 software were used to predict protein subcellular localization. (Horton et al., 2007). The violin plot was constructed using GraphPad Prism 9.3.1 software (GraphPad Inc.,San Diego, California USA, www.graphpad.com)

### Chromosomal localization of *CCoAOMT* genes

Information on the physical location of the *CCoAOMT* sequences from two jute species were collected from the corresponding GFF files, and TBtools (v.1.0971) (Chen et al., 2020) was used to visualize the distribution of *CCoAOMT* genes on each *C. capsularis* and *C. olitorius* chromosome. All the genes involved in the lignin production pathway are highlighted in black, while the *CCoAOMT* genes are highlighted in red. The gene density information was also depicted in the chromosomes.

### Exon-intron distribution of *CCoAOMT* genes, domain architecture along with the amino acid contents in CCoAOMT proteins

The gene structure was created using the Gene Structure Display Server. Pfam was used to obtain domain information for each CCoAOMT protein (Mistry et al., 2020) for the confirmation of identifying domain of this family Methyltransferase-3. The domain architecture was constructed using DOG2.0 software (Ren et al., 2009). The amino acid content was obtained from the Protparam tool of Expasy (Gasteiger et al., 2005).

### Analysis of Cis-regulatory elements

To identify the cis-regulatory element present in the promoter region of two species of Jute, a 2.0 kb upstream region of each gene was used. The potential upstream sequences were submitted to the PlantCARE database (Lescot, 2002) for identification and analysis of several cis-regulatory elements. Later, sum of all elements present in each jute species was visualized as Heatmap constructed using the “Heatmap Illustrator” option in TBtools (v.1.0971) (Chen et al., 2020) software. The total number of different cis-elements in each jute species were categorized into four groups according to their role under various condition (stress, hormone, growth and light) and were visualized as Venn diagram using TBtools (v.1.0971) software (Chen et al., 2020).

### Phylogenetic analysis along with conserved motif

To get an overview of the evolutionary pattern of the *CCoAOMT* gene family in jute species, a phylogenetic tree was constructed using the sequences from *Arabidopsis thaliana*, *Oryza sativa*, *Sorghum bicolor*, *Medicago truncatula*, *Populus trichocarpa*, *Vitis vinifera* and *Camellia sinensis*. The peptide sequences of CCoAOMTs of *Camellia sinensis* were obtained from tea genome database (TIPA, http://tpia.teaplant.org/) (Xia et al., 2019) and the sequences from other species were from Phytozome (https://phytozome.jgi.doe.gov/) (Goodstein et al., 2011). The phylogenetic tree was constructed by IQ-Tree v.2.1.2 (Minh et al., 2020) with 1000 ultrafast bootstrap replicates using the alignment file generated through MAFFT tool v.7 (Katoh et al., 2019) with default parameters. The tree was visualized and edited using iTOL (v.6) (Letunic and Bork, 2019). Information of all the sequences used in this phylogenetic tree are listed in **Supplementary Table 4**. MEME SUITE (v.5.4.1) (Bailey et al., 2009) was used to identify the conserved motif with default parameters except the number of motifs was set to 15 in this case. The information on the conserved motif was uploaded in iTOL (v.6) as an additional dataset along with a phylogenetic tree.

### *In-silico* expression analysis of *CCoAOMT* genes throughout different tissues and analysis of their stress responsive cis-regulatory elements

Multiple sequence alignment of all selected candidate protein sequences was performed using the online version of MAFFT program v.7 (Katoh et al., 2019) with default parameters to determine the sequence conservation and functional homology of CCoAOMTs within the two species. With the multiple sequence alignment files, a phylogenetic tree was generated using 1000 ultrafast bootstrap iterations using IQ-Tree v.2.1.2 (Minh et al., 2020). The tree was then visualized and edited using iTOL (v.6) (Letunic and Bork, 2019). The cis-regulatory elements and *in silico* expression data from different developmental stages were uploaded as an additional dataset in the required format. For the analysis of cis-regulatory elements 2.0 kb regions of each member were analyzed using PlantCARE database (Lescot, 2002).

### Synteny analysis

The whole proteome of both jute species and Arabidopsis were compared using the blastp command of DIAMOND (v2.0.13) with default parameters (Buchfink et al., 2021). The resulting.blast file was used to detect Synteny blocks between each pair of species with the help of were detected using MCScanX (Wang et al., 2012). The blocks were and later visualized using SynVisio (Bandi and Gutwin, 2020).

### RNA extraction and qRT-PCR analysis

The total RNA from the stressed leaf and stem tissues, as well as the control tissue, was extracted using the Monarch Total RNA Miniprep Kit (New England Biolabs, #T2010) according to the manufacturer’s procedure. Following the manufacturer’s protocol, the first strand cDNA was synthesized using a ProtoScript^®^ First Strand cDNA Synthesis Kit (New England Biolabs, #T2010). The expression of each gene was measured with gene-specific primers through qRT-PCR analysis with CFX96 Touch Real-Time PCR Detection System (Bio-Rad, United States) and SYBR Green mixture. The relative expression of the genes was determined using the 2^-ΔΔ Ct^ method (Livak and Schmittgen, 2001), where ΔΔCt was the difference in the threshold cycles and the reference gene, which was *actin* for the expression analyses. The sequences of all gene-specific primers are presented in **Supplementary Table 3**.

### Statistical analysis

Statistical data analysis was performed from three biological replicates under each treatment and time-point (n = 3). Statistical significance was determined using the Analysis of variance (ANOVA) test at P value ≤ 0.05 that were marked with different letters.

## Results

### Histochemical assay of lignin deposition and analysis of accumulation of stress responsive amino acid and phenolics in jute plants under stress

**Figure 1:**
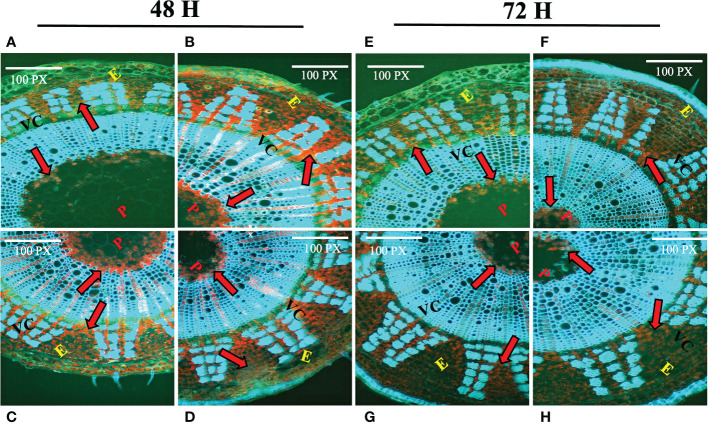
Histochemical assay of lignin deposition in two months old *C olitorius* stem under control and stressed conditions at different time intervals (48 hours and 72 hours). **(A–D)** Stem section of control and three stress treated plants **(A)** control **(B)** hormone **(C)** drought **(D)** salinity stress; after 48 hours; and **(E–H)** Stem section of control plant and three stress treated plants **(E)** control **(F)** hormone **(G)** drought **(H)** salinity stress; after 72 hours. Different letters indicate different regions of the stem (E, epidermis; VC, vascular cambium; P, pith). Red arrows indicate the deposition of lignin.

One of the key components of the jute cell wall, lignin, is expected to play an important role in combating numerous biotic and abiotic stressors. Histochemical assay of lignin was carried out using phloroglucinol. In control plants (**Figures 1A, E**), reddish deposition of lignin was found between the epidermis (E), vascular cambium (VC) and pith (P) region. After 48 hours of stress exposure, the deposition of lignin increased notably in stressed tissues (**Figures 1B–D**) which is evident based on the intensity of the staining. In contrast, 72 hours post-stress, the deposition of lignin was found to be decreased in stressed tissues (**Figures 1F–H**). Apart from that, plants depend on the amino acid proline for several functions. It shields plants from a variety of challenges and also promotes a quicker recovery time for plants after experiencing stress. In response to stress, plants accumulate phenolic compounds as a defensive response. In this study, proline and phenolics accumulation of the farmer’s popular variety, BJRI Tossa Pat-8 variety of *C. olitorius* was analyzed under different abiotic stresses (20 uM ABA, 200 mM NaCl and 20% PEG stress) and proline content was measured after 72 hours in leaf and stem tissues. The amount of proline accumulation was found to be higher in stem tissues compared to leaves and highest accumulation was found under drought stress (**Supplementary Figure S1A**). In contrast, a higher accumulation of phenolics was found in leaf tissues under all stresses (**Supplementary Figure S1B**). Because *CCoAoMT* gene family is engaged in the lignin production pathway, which is also a substantial component of jute stem bark, involvement of this gene family under diverse abiotic stressors will be an important study for selecting prospective gene members for various investigations.

### Identification of *CCoAOMT* genes in jute

**Figure 2:**
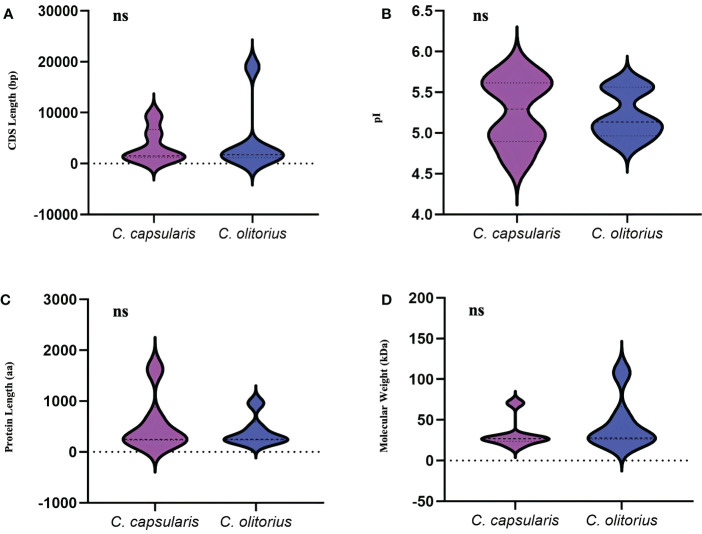
Overview of the physical and chemical properties of the *CCoAOMT* family members in two jute species. **(A)** CDS length of *CCoAOMT* genes and **(B)** Sum of amino acid residues in CCoAOMT proteins. **(C)** The molecular weight of CCoAOMT proteins. **(D)** The isoelectric point of CCoAOMT proteins. The ‘ns’ means no significant difference.

To identify the gene family members responsible for the synthesis of lignin, one of the important lignin bisysnthesis gene, *CCoAOMT* family were studied in jute. A total of six *CCoAOMT* genes were identified in each of the jute species. Genes were named *CcCCoAOMT* and *CoCCoAOMT* for *C. capsularis* and *C. olitorius*, respectively. All details of the identified genes are listed in **Supplementary Table 1**. Genes were numbered 1-6 based on their position on the chromosomes. The CDS length ranged from 1147 bp (*CcCCoAOMT2*) to 9315 bp (*CcCCoAOMT6*) in case of *C. capsularis* and from 1136 bp (*CoCCoAOMT3*) to 18963 bp (*CoCCoAOMT4*) in case of *C. olitorius* (**Supplementary Table 1**). Meanwhile, length of the proteins encoded by these genes ranged from 231 aa (CcCCoAOMT3) to 1624 aa (CcCCoAOMT6) in case of *C. capsularis* and 226 aa (CoCCoAOMT2) to 957 aa (CoCCoAOMT4) in case of *C. olitorius* (**Supplementary Table 1**). No noticeable variations were found in terms of CDS length or amino acid number between the two jute species (**Figure 2**). Additionally, the physical and chemical characteristics of CCoAOMT proteins were examined, and molecular mass and isoelectric points showed no discernible differences (**Figure 2**). Most of the CCoAOMTs were predicted to be localized in cytoplasm except for CcCCoAOMT6 (Nuclear and mitochondrial) and CoCCoAOMT4 (Cytoplasm and Chloroplast) (**Supplementary Table 1**).

### Chromosomal localization of *CCoAOMT* genes

**Figure 3:**
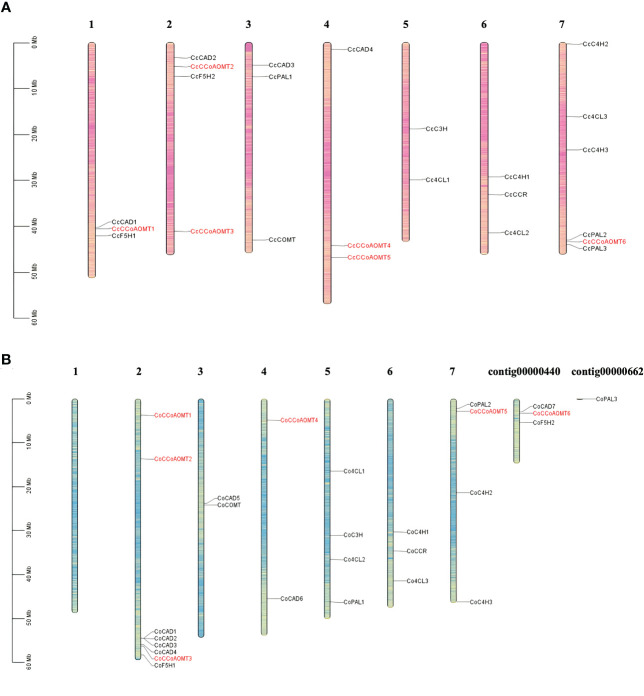
Distribution of the gene members involved in lignin biosynthesis of both *C capsularis* and *C olitorius* species. The *CCoAOMT* genes are indicated in red. Rest of the gene family members are marked in black. Each gene member pointed to the exact position of a specific chromosome, which could be estimated using the scale on the left. The scale in Megabase is represented by the left bar (Mb). Chromosome numbers are provided at the top of each chromosome bar. **(A)** Chromosomal localization of 6 *CcCCoAOMT* genes on 4 *C capsularis* chromosomes. **(B)** Chromosomal localization of 6 *CoCCoAOMT* genes on 3 *C olitorius* chromosomes and 1 contig.

Gene families those are crucial for lignin biosynthesis include- *Phenylalanine ammonia‐lyase (PAL), Cinnamic acid 4‐hydroxylase (C4H), 4‐Coumarate‐coenzyme A ligase (4CL), caffeic acid O‐methyltransferase (COMT), hydroxycinnamoyltransferase (HCT), Cinnamoyl‐CoA reductase (CCR), cinnamyl alcohol dehydrase (CAD), caffeoyl coenzyme A 3‐O‐metyltransferase (CCoAOMT), p‐coumarate 3‐hydroxylase (C3H)* and *Coniferaldehyde 5‐hydroxylase (F5H)* (Yoon et al., 2015). The basic number of chromosome set for two species of jute is n=7. To identify their distribution and position on the jute chromosomes, a chromosome map was generated. All the gene members of the above mentioned families are represented in **Figure 3**. Members of the *CCoAOMT* gene family are indicated in red, whereas other gene members are marked in black. The chromosomal localization of the identified 6 *CCoAOMT* genes of *C. capsularis* reveals that these genes are located in 4 out of the 7 chromosomes (**Figure 3A**), and in *C. olitorius*, all 6 genes are located on 3 chromosomes and 1 contig (**Figure 3B**). Chromosome 1 of *C. olitorius* is devoid of any lignin biosynthesis pathway gene.

### Exon-Intron distribution of *CCoAOMT* genes, domain architecture along with amino acid contents in CCoAOMT proteins

**Figure 4:**
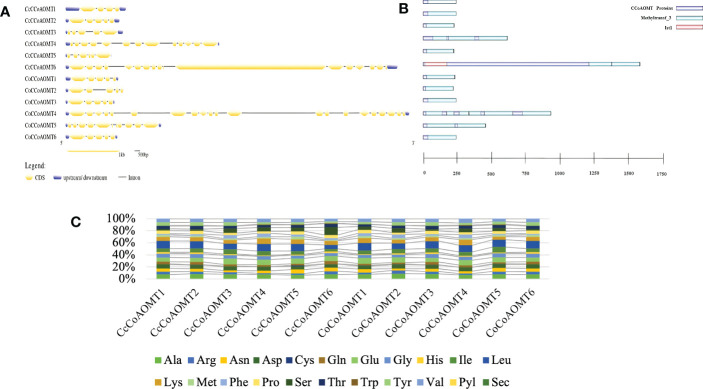
Overview of gene structure, domain architecture and amino acid composition of CCoAOMT members in jute. **(A)** Exon-intron distribution of all *CCoAOMT* genes discovered in *C capsularis* and *C olitorius*. Exon and UTR regions are represented by yellow and green boxes, respectively, while introns are represented by black lines. **(B)** Domain analysis of CCoAOMT proteins from the two species. Green boxes represent the identifying domain Methyltransf_3 and pink box Ist1 domain. **(C)** A stacked bar plot depicting the amino acid (aa) composition of CCoAOMT proteins in both species. Each amino acid’s percentage content is displayed in a different color.

The exon-intron distribution was examined to gain a better understanding of the structure and evolution of the *CCoAOMT* genes. The number of exons and introns in both jute species show little variation. Nine out of a total of twelve members from both species contain 5 exons (**Figure 4A**). The highest number of exons was found in *CoCCoAOMT4*, which is 21 (**Figure 4A**). Variation in the exon-intron distribution indicates the structure of this gene family members is evolutionarily conserved which puts light on their specific role in the lignin biosynthesis pathway. Moreover, all members of this gene family had the characteristic domain Methyltransferase 3, confirming their identity as a member of the *CCoAOMT* gene family. In concordance with the gene structure, the highest number of domains were found in *CoCCoAOMT4* (**Figure 4B**). The percentage of the composition of aa is also found to be highly similar in all 12 CCoAOMT proteins of two jute species (**Figure 4C**). Overall, only minor variations were observed in structural properties between the *CCoAOMT* in these two species.

### Analysis of Cis-regulatory elements

To further understand the putative regulatory networks and the elements responsible for the specific function of the *CCoAOMT* family genes, cis-elements in the 2 kb upstream sequences of the 6 *CCoAOMT* genes of each jute species were evaluated using PlantCARE. There were 74 different types of cis-elements discovered, which were then classified into four categories: stress, hormone, growth and light. The total number of various cis-elements under these four categories ranges from 4 (*CcCCoAOMT1*) to 20 (*CcCCoAOMT2*) in case of abiotic and biotic stress responsive elements, from 6 (*CcCCoAOMT6*) to 15 (*CcCCoAOMT1*) in case of hormone related elements; from 46 (*CcCCoAOMT1*) to 164 (*CcCCoAOMT4*) and from 6 (*CcCCoAOMT3*) to 16 (*CcCCoAOMT4*) in case of growth and light-responsive cis-elements, respectively in *C. capsularis* promoter region. In case of *C. olitorius* promoters, sum of different cis- elements ranges from 2 (*CoCCoAOMT6*) to 15 (*CoCCoAOMT1*) in case of stress-related elements, from 3 (*CoCCoAOMT6*) to 23 (*CoCCoAOMT1*); from 46 (*CoCCoAOMT6*) to 116 (*CoCCoAOMT3*) and from 4 (*CoCCoAOMT6*) to 20 (*CoCCoAOMT1*) in case of hormone, growth and light-responsive cis-elements, respectively. The lignin-associated element name Myb element (Patzlaff et al., 2003) was found in all the members except for *CcCCoAOMT1* and *CoCCoAOMT6*.

### Phylogenetic analysis along with conserved motif

**Figure 5:**
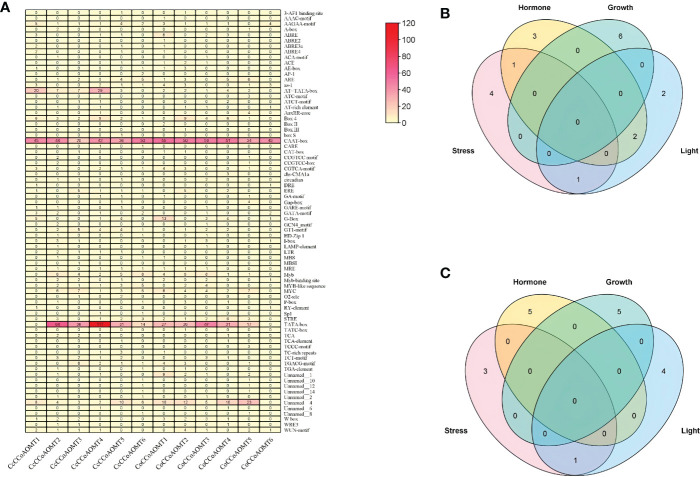
Study of the cis-regulatory elements present in the 2kb upstream putative promoter region of *CCoAOMT* genes in jute. **(A)** Total number of different cis-elements present in two species of Jute. The total number of cis-elements in each *CCoAOMT* gene of *C capsularis* **(B)** and *C olitorius CCoAOMT* in four categories is depicted by Venn diagram **(C)**.

**Figure 6:**
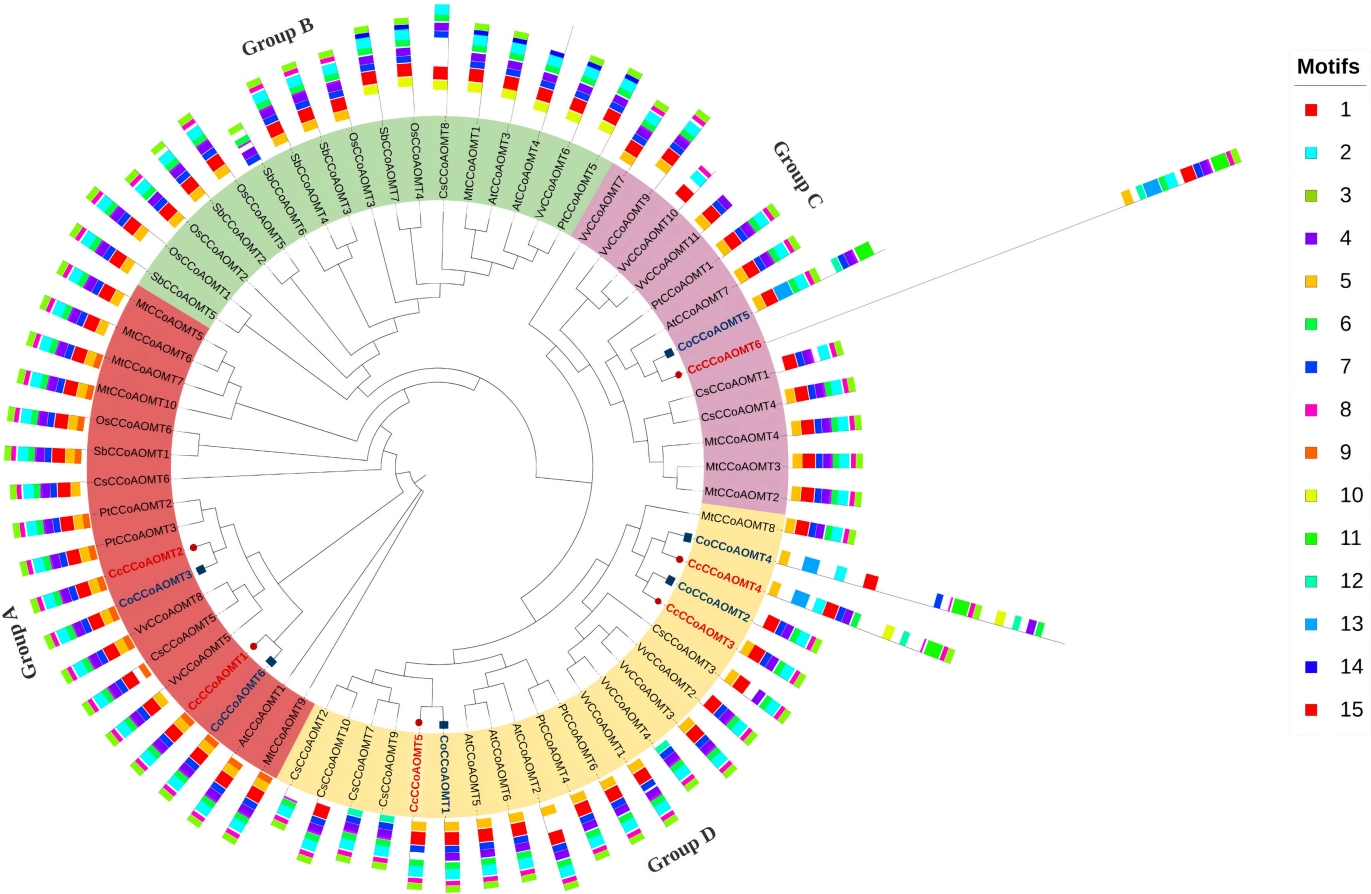
Phylogenetic relationship of CCoAOMTs of different plant species along with conserved motifs. A maximum likelihood method using 1000 ultrafast bootstrap replicates was used to construct the tree. The CCoAOMTs of *C. capsularis* and *C. olitorius* are labeled in red. The phylogenetic tree clustered into four major groups depicted in different colors. The 15 identified conserved motif is also added as an additional dataset represented in different colored boxes.

To analyze the evolutionary pattern, a phylogenetic tree was constructed based on a total of 69 CCoAOMT proteins, including 6 from *C. capsularis*, 6 from *C. olitorius*, 10 from *C. sinensis*, 7 from *A. thaliana*, 6 from *O. sativa*, 7 from *S. bicolor*, 10 from *M. truncatula*, 6 from *P. trichocarpa*, and 11 from *V. vinifera*. The CCoAOMTs are clustered into four groups in the phylogenetic tree (Group A, B, C and D). Group A contains two CCoAOMTs from *C. capsularis* and two from *C. olitorius* where most of the CCoAOMTs are associated with lignin biosynthesis. Except for Group D, the other three groups contain CCoAOMT proteins from two jute species. In concordance with **Figure 5**, the same members from *C. capsularis* and *C. olitorius* clustered into the same branches in this phylogenetic tree. To explore the difference among the protein structures, the MEME website was used to examine the variations in protein structures. Typically, most of the closely related individuals within a group share similar motif compositions (**Figure 6**). Motifs 1, 2, 3, 4 and 6 were found to be the most prevalent in all CCoAOMT proteins except for a few. Some motifs were exclusively found in a certain group in the phylogenetic tree (Motif 10, 11, 13 and 14). These unique motifs may contribute to the distinct roles of CCoAOMT proteins. In the case of the CCoAOMT proteins of *C. olitorius* and *C. capsularis*, motifs 1, 2 and 4 were found in all the members.

### *In-silico* expression analysis of *CCoAOMT* genes in different tissues and analysis of their stress responsive cis-regulatory elements

**Figure 7:**
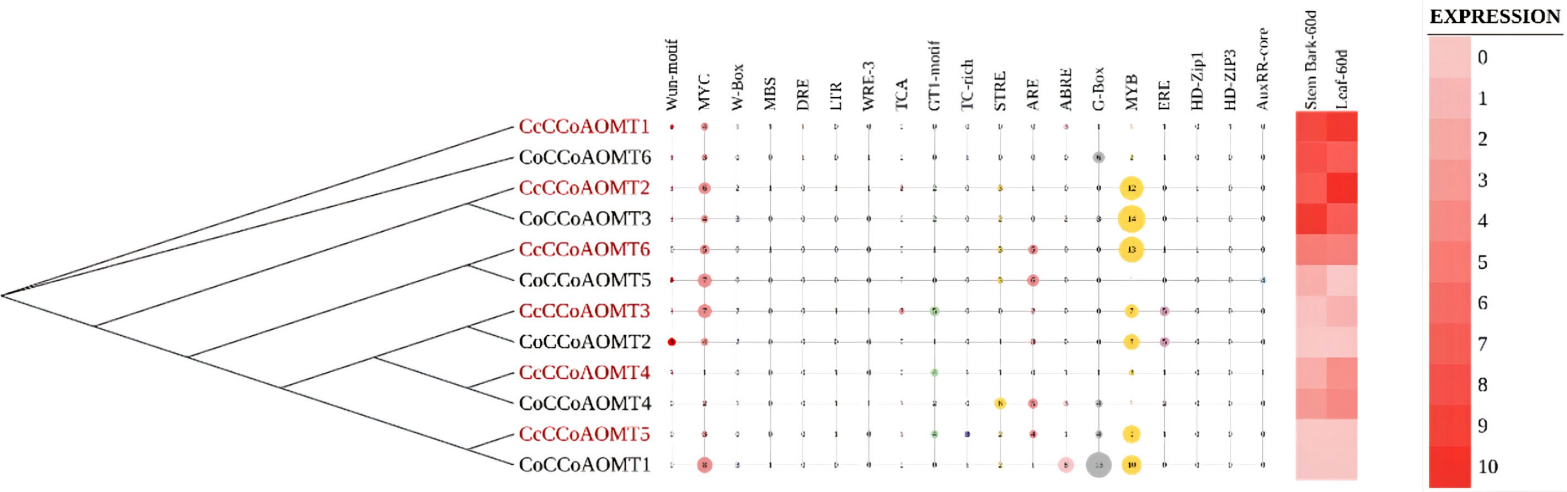
Phylogenetic relationship among two jute species along with their different stress-responsive cis-elements to demonstrate their regulatory response upon stress and *in-silico* expression profiles of different tissues (60 days old stem bark and leaf).

To decipher the role of *CCoAOMT* genes their expression profile in two plant parts- stem bark and leaf, were analyzed along with the presence of different cis-elements in two jute species. The phylogenetic analysis of *CCoAOMT* genes across multiple plant species showed that genes with similar functions from different species cluster together in the same clade. Here, gene members with high expression values are clustered together in the phylogenetic tree. One of the stress-responsive cis-elements, Myb showed the most frequent abundance among the gene family members with higher expression that might function in the biosynthesis of lignin in plants. The number of Myb regulatory elements was found more prevalent in highly expressive genes, and genes with low expression showed minimum presence of Myb element. Presence of other elements could not be directly correlated with the expression data of 12 genes in stem bark and leaf (**Figure 7**).

### Synteny between the two jute genomes

**Figure 8:**
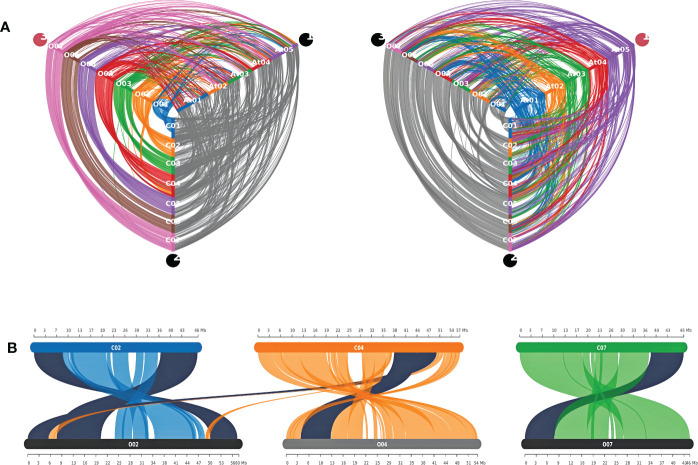
Synteny analysis between *C capsularis*, *C olitorius*, and *A thaliana* genomes. **(A)** Hive plots showing the syntenic conservation among the three genomes. **(B)** Syntenic *CCoAOMT* genes between the two jute genomes. Synteny pairs are highlighted in black.

A synteny analysis was carried out to get an overview of the similarities between the two jute genomes. Expectedly, the results show that there is a high degree of syntenic conservation between the two genomes. In almost all cases, each chromosome in one species is seen to be in high synteny with a similar chromosome in the other species. However, all chromosomes except Chr06, show evidence of chromosomal inversion (**Figure 8A** and **Supplementary Figure S2**). Moreover, in case of Chr03, there appear to be two separate incidences of inversion. In contrast to this, when compared to the *A. thaliana* genome, both jute species exhibit less synteny; with the syntenic regions being much smaller than the regions between the two jute genomes (**Figure 8A**). Furthermore, for each jute chromosome, the synteny blocks are dispersed among all five *A. thaliana* chromosomes. This points to a higher number of genomic rearrangements throughout the evolutionary history between the two jute species and *A. thaliana*.

As previously mentioned, all the methyltransferase genes in this study were distributed over four chromosomes in both jute species (**Figure 3**). Out of the six genes, five were found to be present in synteny blocks, with one-to-one conservation between the two jute genomes (**Figure 8B** and **Supplementary Table 2**). Interestingly, one of the *C. capsularis* genes (*CcCCoAOMT5*) appears to be a part of a syntenic block that extends from Chr04 to Chr02 of *C. olitorius*. This likely indicates a translocation event at this genomic region, leading to both homologous genes (*CcCCoAOMT5* and *CoCCoAOMT1*) being located on different chromosomes.

### Expression profiling of *CoCCoAOMT* gene family under hormone, drought and salinity stresses

**Figure 9:**
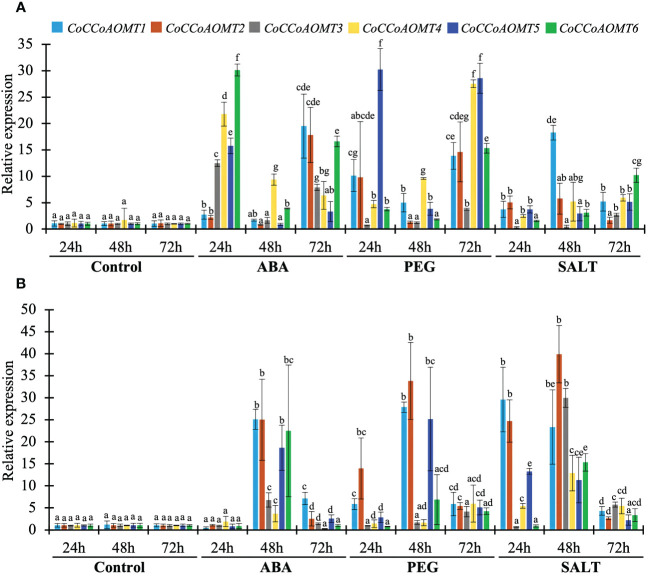
Expression profiles of *CoCCoAOMT* genes against control, hormonal, drought, and saline conditions. The left margin scale depicts the relative expression of *CoCCoAOMT* gene under control and various stress conditions. The results were reported as the mean value of the relative expression with standard deviation, using the jute Actin gene as a reference gene. Relative expression of *CoCCoAOMT* gene in *C olitorius* under control, ABA, PEG, and NaCl treatments in leaf **(A)** and stem **(B)** tissue. The expression level of each *CoCCoAOMT* gene at all three times under control condition was set to 1. Columns at each observation and time point followed by the different letters (such as a, b, c, d, e, f, or g) are statistically significantly different using the Analysis of variance test at P value ≤ 0.05, while columns with the same letter are statistically not significant (P value > 0.05).

As the level of lignin is altered in response to different abiotic stresses (**Figure 1**), it is crucial to assess the expression of all 6 *CoCCoAOMT* genes experimentally for correlation purpose. Thus, we carried out quantitative RT-PCR analysis of 6 *CoCCoAOMT* genes in leaf and stem tissues after hormonal treatment of abscisic acid (ABA), polyethylene glycol (PEG) and 200mM NaCl for 24h, 48h and 72h. Most of the genes are found to be upregulated in response to all three conditions compared with respective control samples with few exceptions (**Figure 9**). Most of the analyzed genes showed significant upregulation in response to ABA treatment at both 24h and 72h in leaf samples with a fluctuating pattern in 48h. An opposite pattern was observed in the stem samples where all 6 genes showed clear upregulation in 48h of ABA treatment. Consistently, most of the genes were upregulated under PEG and NaCl treatment in all three-time points. Interestingly, the transcript abundance of all 6 genes was found to be decreased gradually in response to salinity in the stem samples.

## Discussion

Lignification is one of the key processes in higher plants that results in the deposition of lignin in the plant cell wall, which protects the plant against numerous biotic and abiotic agents (Cesarino, 2019) Several studies have found that after 12 days of water retention, decreased anionic peroxidase activity and increased cationic peroxidase activity, as well as an increase in free lignin precursors in the xylem sap, could be an indication that water stress reduces lignin biosynthesis in maize (Alvarez et al., 2008). Although various tissues in different places may behave differently under stress; for instance, specific regions of maize roots may react distinctly to water deficit (Fan et al., 2006; Yang et al., 2006; Yoshimura et al., 2008). To understand the lignin deposition status in jute plant, a histochemical assay of lignin was performed in this study for both control and stressed plants (post 48 and 72 hours) (**Figure 1**). The stain intensity suggests that the deposition of lignin increased after 48 hours of stress exposure but decreased after the stress has been induced for a longer time i.e., 72 hours. As multiple gene families are involved in the lignin synthesis of plants, the relative expression of specific genes involved in the pathway might influence the ultimate lignification of jute under stress conditions. Along with the secondary cell wall component lignin, quantity of certain amino acids and secondary metabolites also found to be increased under stress conditions. The accumulation of proline and phenolics in stressed jute plants were measured and found to be accumulated significantly in response to stress conditions compared with untreated control sample (**Supplementary Figure 1**). Proline causes plants under stress to develop more quickly and exhibit other positive physiological traits when administered exogenously to the plants. In addition to that, plants play a crucial function in the management of diverse environmental stresses and accumulate phenolic compounds in their tissues as an adaptive response to unfavorable environmental conditions (Naikoo et al., 2019). Here, higher deposition of lignin and high accumulation of proline and phenolic compounds was found in stressed plants which confirms the plants response mechanism under abiotic stresses.

In this study, we focused on the gene members involved in the formation of lignin in jute plants. The *CCoAOMT* gene family is one of the least investigated of the many gene families involved in the production of lignin in plants. Discovering the number and role of genes involved in the manufacture of lignin, which impacts the quality of fibers, is critical for fiber bearing crops such as jute. Here, two species of jute (*C. capsularis* and *C. olitorius*) were investigated to uncover the abundance, structure, relative expression and role of the *CCoAOMT* gene family that is involved in lignin biosynthesis in plants. The *CCoAOMT* gene family has been previously studied in several plants, including *A. thaliana, S. bicolor, P. trichocarpa, V. vinifera*, and *C. sinensis*, with members of the gene family reported to be 7, 7, 6, 11, and 10; respectively (Raes et al., 2003; Hamberger et al., 2007; Giordano et al., 2016; Rakoczy et al., 2018; Lin et al., 2020). According to previous reports, the *CCoAOMT* gene family consists primarily of fewer family members in various plant species. We found both the species of jute were found to contain 6 *CCoAOMT* genes each which is in harmony with previous report.

In terms of the physical features and molecular attributes of the *CCoAOMT* genes these two jute species showed very little variation which indicates the functional conservation of this gene family in both jute species. Synteny analysis also revealed great conservation between the two jute genomes with an incidence of chromosomal inversion. Most of the *CCoAOMT* genes were found to have a one-to-one ortholog in the two species. Although the products of lignin biosynthesis and other phenylpropanoid routes are generally found in vacuoles and the cell wall, the pathways themselves are found in the cytoplasm, suggesting that the enzymes involved in the processes may be found there as well (Hrazdina and Jensen, 1992; Schmitt and An, 2017). Previous research has demonstrated that CCoAOMTs are cytoplasmic localization proteins (Chen et al., 2000; Zhang and Erickson, 2012) which aligns with our present result. Cis-Regulatory elements in promoter regions regulate the function of specific genes by binding to the right transcription factors (Hernandez-Garcia and Finer, 2014). For this reason, cis-elements in promoter regions are crucial for controlling gene expression. As a result, the role of *CCoAOMT* genes in lignin biosynthesis was explored by investigating the cis-acting regulatory components related to lignin biosynthesis in the promoter regions of *CCoAOMT*s. Studies suggest that by interacting with the P-box element, the Myb Plant motif found in the promoters of genes involved in phenylpropanoid biosynthesis controls the production of lignin (Tamagnone et al., 1998). Except for *CcCCoAOMT1* and *CoCCoAOMT6*, all of the genes in our investigation had the lignin-associated element Myb (Patzlaff et al., 2003) in their promoter region, indicating that these members are involved in the production of lignin. In addition, all cis-elements were divided into four categories: those that relate to growth, hormones, stress, and light. The role played by this gene family in each of these four sectors is evidenced by the prevalence of cis elements in all four of these categories. The presence of various stress-responsive elements, such as LTR (low temperature), MBS (drought), TC-rich repeats (stress and defense), WUN-motif (wound), etc., were also found, which is consistent with the earlier study on *CCoAOMTs* in tea (Lin et al., 2020). Moreover, some of the highly expressed members (*CcCCoAOMT2, CcCCoAOMT6* and *CoCCoAOMT3*) contain a higher number of Myb elements in their respective promoter region (**Figure 7**). It could be assumed that these members are actively involved in the deposition of lignin due to their higher expression in the lignin depositing tissues. The role of these genes in the production of secondary cell wall components and in protecting plants from various biotic and abiotic stressors need to be studied further.

Based on phylogenetic analysis of 69 CCoAOMT protein sequences from jute plants and other species, CCoAOMTs can be clustered into four groups. No CCoAOMT proteins of jute plants were found in group B. CcCCoAOMT1, CcCCoAOMT2, CoCCoAOMT3 and CoCCoAOMT6 proteins clustered into group A which contains the proteins that are involved in lignin biosynthesis pathway (Guo et al., 2001; Day et al., 2009; Lin et al., 2020). Group A also contains AtCCoAOMT1 protein, which is considered the true CCoAOMT gene, while the other classes were considered CCoAOMT-like genes (Rakoczy et al., 2018). Proteins clustered in the same clade generally exhibit similar functions. Thus, CcCCoAOMT1, CcCCoAOMT2, CoCCoAOMT3 and CoCCoAOMT6 may play major roles in lignin biosynthesis. Similar motif patterns were also found in the same groups of the phylogenetic tree (**Figure 6**) which further confirms their function in the biosynthesis of lignin. In concordance with **Figure 6**, the phylogenetic tree within the jute species also showed clustering of CcCCoAOMT1, CoCCoAOMT6 and CcCCoAOMT2, CoCCoAOMT3 together in the same branch. The *in-silico* expression value of these four members was also found high in stem bark and leaf tissues. These results further put light on the importance of these gene members in transcriptional modulation of the *CCoAOMT* gene family in jute. To validate the *in-silico* expression result and to elucidate the expression profile of the *CCoAOMT* gene family under different abiotic stresses (hormone, drought and salinity), two months old *C. olitorius* seedling was examined. Surprisingly, leaf and stem tissues showed a differential expression patterns. In leaf tissues, most of the gene members showed up-regulation in 24- and 72-hours time points, whereas in stem tissues most genes were up-regulated after 48 hours of stress exposure. The reason behind this, in fiber crops most of the lignin is deposited in the stem tissues and when the plants are exposed to sudden stress shock, most of the lignin and other secondary metabolites work to combat the stress. During this process, the expression level of *CoCCoAOMTs* in stem tissues increased gradually from 24 hours up to 48 hours of stress. After a certain period, the expression level again reduces, as there is a certain trade-off between the growth and defense mechanism in plants (Ha et al., 2021). Contrarily, under stress conditions, leaf tissues readily act to fight the stresses and gradually decreases their expression to focus on growth. But during long exposure to stress, the expression level was again found to be increased in leaf tissues. From the expression profile of *CoCCoAOMTs* under stress, some members were more upregulated than others. According to Rakoczy et al., 2018, clade 1 characteristics are demonstrated by the high expression of *SbCCoAOMT1* in leaves and stems with high lignification. In plants, the down-regulation of *CCoAOMT* results in a striking reduction in lignin content, indicating *CCoAOMT* is involved in the up-regulation of lignin production (Jianhua et al., 2001; Zhao et al., 2002). In the *in-silico* expression analysis, two members- *CoCCoAOMT3* and *CoCCoAOMT6* were found to be highly expressive in two important tissues- stem and leaf (**Figure 7**). Similarly, both *CoCCoAOMT3* and *CoCCoAOMT6* transcripts showed significant upregulation under all three stresses in both leaf and stem tissues (**Figure 9**). Apart from these two, *CoCCoAOMT4* and *CoCCoAOMT5* showed significant upregulation in leaf tissues; and *CoCCoAOMT1* and *CoCCoAOMT2* in stem samples in response to all three stresses. Taken together, this study confirms the function of the *CCoAOMT* gene family members in the lignin biosynthesis as well as stress modulation pathways of jute. Therefore, further efforts should be made to overexpress or silence jute *CCoAOMT* genes using a transgenic technique employing *Arabidopsis* or other model plants to understand their molecular functions.

## Conclusion

Overall, this study provides a thorough evaluation of the *CCoAOMT* gene family in the fiber crop jute on a genome wide scale. We were able to locate 6 *CCoAOMT* genes from two jute species in total, which is in harmony with previous studies. This study sheds light on *CCoAOMT* gene regulation under various stresses in jute. The multi-stress responsiveness of the prospective jute *CCoAOMT* genes was also confirmed by qRT-PCR. However, further study is needed to determine the functional role of these genes by creating overexpressing or knockout lines of *CCoAOMT* genes individually or collectively. This study will facilitate further investigation of the *CCoAOMT* gene family in jute and other plants.

## Data availability statement

The original contributions presented in the study are included in the article/supplementary materials. Further inquiries can be directed to the corresponding author.

## Author contributions

TI conceived the idea and designed the experiments. SA, AS and TT performed all the experiments. TI, SA and AS analysed of data. SA and AS wrote the manuscript. TT and BJ helped in preparing manuscript. All authors contributed to the article and approved the submitted version.

## Supplementary material

Supplementary Figure 1 Proline and phenolic content in *C. olitorius* in response to abiotic stress were measured. The accumulation was assessed in two-month-old jute seedlings under various stress conditions, such as salt (200 mM NaCl), drought (20% PEG), and hormone (20 uM ABA), as well as related control samples. The mean value and standard deviation (n = 3) were used to represent the results. **(A)** proline content in leaf and stem tissue under stress. **(B)** phenolic content in leaf and stem tissue in response to stress. Columns followed by the different letters are statistically significantly different according to P value ≤ 0.05.

Supplementary Figure 2 Syntenic relationship between two jute genomes (*C. capsularis* and *C. olitorius).*
