# Supplementary figures and images for: Caffeoyl-CoA 3-O-methyltransferase gene family in jute: Genome-wide identification, evolutionary progression and transcript profiling under different quandaries
Source: Front Plant Sci. 2022 Dec 14;13:1035383. doi: 10.3389/fpls.2022.1035383 (PMC9798919; doi:10.3389/fpls.2022.1035383)

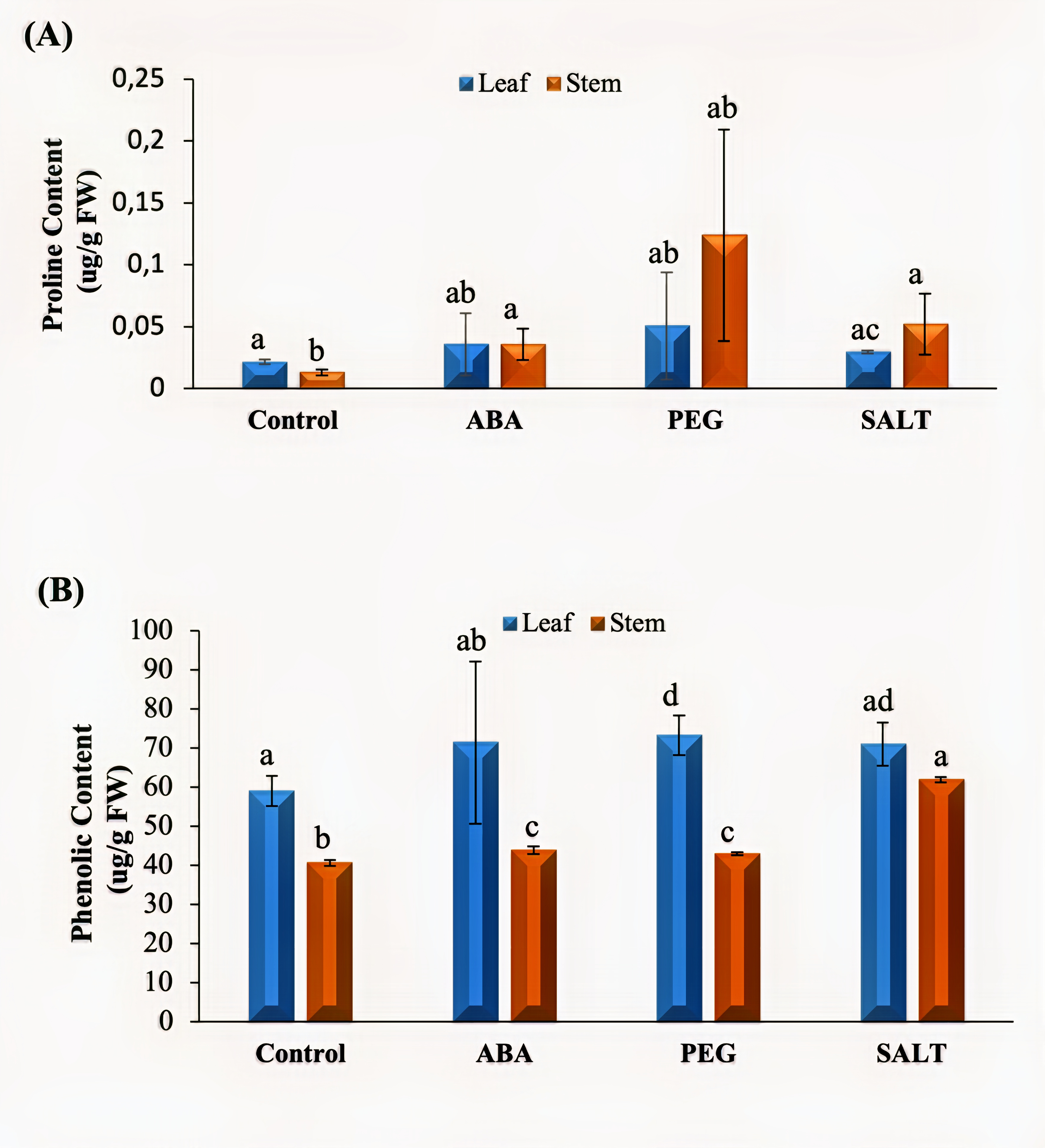

Supplement: Supplementary Figure 1 — Proline and phenolic content in C. olitorius in response to abiotic stress were measured. The accumulation was assessed in two-month-old jute seedlings under various stress conditions, such as salt (200 mM NaCl), drought (20% PEG), and hormone (20 uM ABA), as well as related control samples. The mean value and standard deviation (n = 3) were used to represent the results. (A) proline content in leaf and stem tissue under stress. (B) phenolic content in leaf and stem tissue in response to stress. Columns followed by the different letters are statistically significantly different according to P value ≤ 0.05. [file Image_1.jpeg]

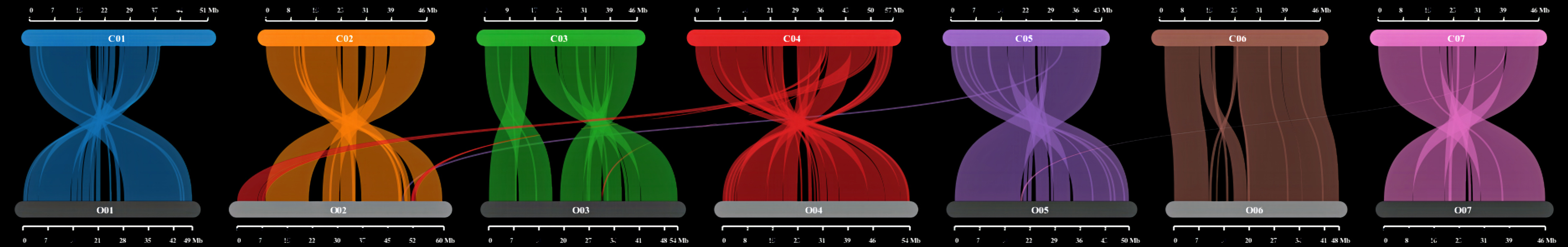

Supplement: Supplementary Figure 2 — Syntenic relationship between two jute genomes (C. capsularis and C. olitorius). [file Image_2.jpeg]
