# Supplementary material for: Caffeoyl-CoA 3-O-methyltransferase gene family in jute: Genome-wide identification, evolutionary progression and transcript profiling under different quandaries
Source: Front Plant Sci. 2022 Dec 14;13:1035383. doi: 10.3389/fpls.2022.1035383 (PMC9798919; doi:10.3389/fpls.2022.1035383)
Supplement: Supplementary file 3 [file DataSheet_1.docx]

## **Supplementary materials and methods**

## **Estimation of proline and phenolic content in stresses jute tissues**

## For the extraction and estimation of proline, fresh leaf and stem tissues were homogenized in 5ml of 0.1M sulphosalicylic acid. Homogenate was centrifuged at 10,000 g for 15 min and subsequently, 2 ml filtrate was mixed with 5 ml of acid ninhydrin and 5 ml of glacial acetic acid. The mixture was incubated in a water bath at 100 8C for 1 h until the colored complex was developed and the reaction was terminated by cooling in ice. The reaction mixture was vortexed with 10 ml of toluene for 15–20 s. The optical density of the layer with chromophore was read at 520 nm. The proline concentration was determined using a standard curve and calculated on a fresh weight basis (usually expressed as microgram per gram FW or micromole per gram FW).

## In case of the extraction of phenolic compounds from stressed jute plants, fresh tissues were homogenized with 10 ml 80% ethanol following centrifugation for 20 minutes at 8000 rpm. Supernatant was taken and sample was re-centrifuged again for 20 minutes. The two supernatants were then evaporated to dryness in a small beaker (sand bath). The residues were then dissolved into 5 ml distilled water (D.W). 3 ml of the extracts was collected and 0.5 ml of folin-phenol reagent was added to this. After 3 minutes, 2 ml of 20% sodium carbonate solution was mixed thoroughly. The mixture was then placed in a water bath for 1 minute followed by cooling in test tubes. The absorbance of the sample was measured at 650 nm against a blank reagent. Likewise, the phenolic concentration was determined using a standard curve and calculated on a fresh weight basis (usually expressed as microgram per gram FW or micromole per gram FW).
