# Supplementary material for: Caffeoyl-CoA 3-O-methyltransferase gene family in jute: Genome-wide identification, evolutionary progression and transcript profiling under different quandaries
Source: Front Plant Sci. 2022 Dec 14;13:1035383. doi: 10.3389/fpls.2022.1035383 (PMC9798919; doi:10.3389/fpls.2022.1035383)
Supplement: Supplementary file 4 [file Table_1.docx]

# **Supplementary Tables**

### **Supplementary Table 1. List of CCoAOMT genes along with their molecular attributes**

| **Gene** | **Locus ID** | **Chr No** | **CDS coordinate (5’ to 3’)** | **CDS (bp)** | **PP (aa)** | **Mass (kDa)** | **pI** | **Localization** |
| --- | --- | --- | --- | --- | --- | --- | --- | --- |
| CcCCoAOMT1 | Cc.01G0029320 | 1 | 40556997-40558187 | 1190 | 247 | 27.92108 | 5.57 | Cyt1,2 |
| CcCCoAOMT2 | Cc.02G0006600 | 2 | 5211059-5212206 | 1147 | 247 | 27.82503 | 5.56 | Cyt1,2 |
| CcCCoAOMT3 | Cc.02G0026850 | 2 | 41098036-41099806 | 1770 | 231 | 26.144 | 4.67 | Cyt1,2 |
| CcCCoAOMT4 | Cc.04G0034440 | 4 | 44184486-44190276 | 5790 | 631 | 70.85558 | 4.97 | Cyt1,2 |
| CcCCoAOMT5 | Cc.04G0037240 | 4 | 46735527-46736796 | 1269 | 229 | 25.75772 | 5.03 | Cyt1,2 |
| CcCCoAOMT6 | Cc.07G0031060 | 7 | 43343400-43352715 | 9315 | 1624 | 18.01075 | 5.75 | Nu1, Mt2 |
| CoCCoAOMT1 | Co.02G0004230 | 2 | 3748993-3750263 | 1270 | 237 | 26.68386 | 4.99 | Cyt1,2 |
| CoCCoAOMT2 | Co.02G0012630 | 2 | 13701853-13704098 | 2245 | 226 | 25.707 | 5.13 | Cyt1,2 |
| CoCCoAOMT3 | Co.02G0039610 | 2 | 56277841-56278977 | 1136 | 247 | 27.82503 | 5.56 | Cyt1,2 |
| CoCCoAOMT4 | Co.04G0005980 | 4 | 4892365-4911328 | 18963 | 957 | 108.0694 | 5.14 | Cyt1, Chl2 |
| CoCCoAOMT5 | Co.07G0003500 | 7 | 2808139-2810754 | 2615 | 468 | 52.364 | 4.89 | Cyt1,2 |
| CoCCoAOMT6 | Co.v20117010 | Contig00000440 | 3271167-3272362 | 1195 | 247 | 27.92108 | 5.57 | Cyt1,2 |

**1WoLFPSORT, 2CELLO subcellular localization prediction tool**

**Mt, mitochondria; chl, chloroplast; Nu, nucleus; Cyt, cytoplasm.**

### **Supplementary Table 2. Synteny gene pairs between *C. capsularis* and *C. olitorius* genomes.**

| **Serial** | ***C. capsularis* chromosome** | ***C. capsularis* gene** | ***C. olitoius* chromosome** | ***C. olitorius* gene** |
| --- | --- | --- | --- | --- |
| 1 | 2 | CcCCoAOMT2 (Cc.02G0006600) | 2 | CoCCoAOMT3 (Co.02G0039610) |
| 2 | 2 | CcCCoAOMT3 (Cc.02G0026850) | 2 | CoCCoAOMT2 (Co.02G0012630) |
| 3 | 4 | CcCCoAOMT4 (Cc.04G0034440) | 4 | CoCCoAOMT4 (Co.04G0005980) |
| 4 | 4 | CcCCoAOMT5 (Cc.04G0037240) | 2 | CoCCoAOMT1 (Co.02G0004230) |
| 5 | 7 | CcCCoAOMT6 (Cc.07G0031060) | 7 | CoCCoAOMT5 (Co.07G0003500) |

**Supplementary Table 3. Primer sequences used in RT-PCR**

| Serial | Gene | Primer sequence |
| --- | --- | --- |
| 01 | CoCCoAOMT1- Forward | GTACCAGTAGACGAAGGGCA |
|  | CoCCoAOMT1- Reverse | ATCATCAGGCAAAGCAAGGG |
| 02 | CoCCoAOMT2- Forward | GCCATGAGTTTGCCTGTTGA |
|  | CoCCoAOMT2- Reverse | CATCCTCAGGCAAAGCAAGG |
| 03 | CoCCoAOMT3- Forward | CTTGCTCTGCCTGAAGATGG |
|  | CoCCoAOMT3- Reverse | GCAGGGCCTTCTTTGAAGTC |
| 04 | CoCCoAOMT4- Forward | GTCGTTGAAGCCACATCCTC |
|  | CoCCoAOMT4- Reverse | TTGTCTGAACTGCTTGGGGA |
| 05 | CoCCoAOMT5- Forward | AGAGGCGGTTTCAGAAGACA |
|  | CoCCoAOMT5- Reverse | GCAAGAGAGATTTCCAGGCG |
| 06 | CoCCoAOMT6- Forward | GAACTCAACAAGGCTCTCGC |
|  | CoCCoAOMT6- Reverse | TTTGATTCGACGGCAAAGGG |

**Supplementary Table 4. The CCoAOMT gene family members in all species used in phylogenetic tree**

| **Species** | **Gene name** | **Gene ID** | **Protein length (aa)** |
| --- | --- | --- | --- |
| *Corchorus capsularis* | *CcCCoAOMT1* | Cc.01G0029320 | 247 |
| *Corchorus capsularis* | *CcCCoAOMT2* | Cc.02G0006600 | 247 |
| *Corchorus capsularis* | *CcCCoAOMT3* | Cc.02G0026850 | 231 |
| *Corchorus capsularis* | *CcCCoAOMT4* | Cc.04G0034440 | 631 |
| *Corchorus capsularis* | *CcCCoAOMT5* | Cc.04G0037240 | 229 |
| *Corchorus capsularis* | *CcCCoAOMT6* | Cc.07G0031060 | 1624 |
| *Corchorus olitorius* | *CoCCoAOMT1* | Co.02G0004230 | 237 |
| *Corchorus olitorius* | *CoCCoAOMT2* | Co.02G0012630 | 226 |
| *Corchorus olitorius* | *CoCCoAOMT3* | Co.02G0039610 | 247 |
| *Corchorus olitorius* | *CoCCoAOMT4* | Co.04G0005980 | 957 |
| *Corchorus olitorius* | *CoCCoAOMT5* | Co.07G0003500 | 468 |
| *Corchorus olitorius* | *CoCCoAOMT6* | Co.v20117010 | 247 |
| *Arabidopsis thaliana* | *AtCCoAOMT1* | AT4G34050 | 259 |
| *Arabidopsis thaliana* | *AtCCoAOMT2* | AT1G24735 | 240 |
| *Arabidopsis thaliana* | *AtCCoAOMT3* | AT3G61990 | 290 |
| *Arabidopsis thaliana* | *AtCCoAOMT4* | AT3G62000 | 278 |
| *Arabidopsis thaliana* | *AtCCoAOMT5* | AT1G67990 | 233 |
| *Arabidopsis thaliana* | *AtCCoAOMT6* | AT1G67980 | 232 |
| *Arabidopsis thaliana* | *AtCCoAOMT7* | AT4G26220 | 232 |
| *Oryza sativa* | *OsCCoAOMT1* | LOC_Os08g38910 | 292 |
| *Oryza sativa* | *OsCCoAOMT2* | LOC_Os08g38920 | 234 |
| *Oryza sativa* | *OsCCoAOMT3* | LOC_Os08g38900 | 252 |
| *Oryza sativa* | *OsCCoAOMT4* | LOC_Os08g05790 | 317 |
| *Oryza sativa* | *OsCCoAOMT5* | LOC_Os09g30360 | 258 |
| *Oryza sativa* | *OsCCoAOMT6* | LOC_Os06g06980 | 260 |
| *Sorghum bicolor* | *SbCCoAOMT1* | Sobic.010G052200 | 261 |
| *Sorghum bicolor* | *SbCCoAOMT2* | Sobic.002G242300 | 241 |
| *Sorghum bicolor* | *SbCCoAOMT3* | Sobic.007G218700 | 250 |
| *Sorghum bicolor* | *SbCCoAOMT4* | Sobic.007G218800 | 246 |
| *Sorghum bicolor* | *SbCCoAOMT5* | Sobic.007G218500 | 267 |
| *Sorghum bicolor* | *SbCCoAOMT6* | Sobic.007G217200 | 144 |
| *Sorghum bicolor* | *SbCCoAOMT7* | Sobic.007G043200 | 306 |
| *Populus trichocarpa* | *PtCCoAOMT1* | POPTR_0018s06630 | 235 |
| *Populus trichocarpa* | *PtCCoAOMT2* | POPTR_0009s10270 | 247 |
| *Populus trichocarpa* | *PtCCoAOMT3* | POPTR_0001s31220 | 247 |
| *Populus trichocarpa* | *PtCCoAOMT4* | POPTR_0010s11500 | 234 |
| *Populus trichocarpa* | *PtCCoAOMT5* | POPTR_0002s18450 | 352 |
| *Populus trichocarpa* | *PtCCoAOMT6* | POPTR_0008s13600 | 235 |
| *Vitis vinifera* | *VvCCoAOMT1* | VIT_201s0010g03490 | 241 |
| *Vitis vinifera* | *VvCCoAOMT2* | VIT_201s0010g03460 | 211 |
| *Vitis vinifera* | *VvCCoAOMT3* | VIT_201s0010g03510 | 235 |
| *Vitis vinifera* | *VvCCoAOMT4* | VIT_201s0010g03470 | 187 |
| *Vitis vinifera* | *VvCCoAOMT5* | VIT_203s0063g00140 | 242 |
| *Vitis vinifera* | *VvCCoAOMT6* | VIT_215s0048g02200 | 280 |
| *Vitis vinifera* | *VvCCoAOMT7* | VIT_212s0028g03110 | 243 |
| *Vitis vinifera* | *VvCCoAOMT8* | VIT_207s0031g00350 | 247 |
| *Vitis vinifera* | *VvCCoAOMT9* | VIT_211s0016g02600 | 292 |
| *Vitis vinifera* | *VvCCoAOMT10* | VIT_211s0016g02592 | 172 |
| *Vitis vinifera* | *VvCCoAOMT11* | VIT_211s0016g02586 | 135 |
| *Medicago truncatula* | *MtCCoAOMT1* | Medtr2g070410 | 298 |
| *Medicago truncatula* | *MtCCoAOMT2* | Medtr8g021225 | 235 |
| *Medicago truncatula* | *MtCCoAOMT3* | Medtr8g101900 | 235 |
| *Medicago truncatula* | *MtCCoAOMT4* | Medtr6g091680 | 238 |
| *Medicago truncatula* | *MtCCoAOMT5* | Medtr4g094925 | 243 |
| *Medicago truncatula* | *MtCCoAOMT6* | Medtr4g094812 | 246 |
| *Medicago truncatula* | *MtCCoAOMT7* | Medtr4g094898 | 248 |
| *Medicago truncatula* | *MtCCoAOMT8* | Medtr4g094368 | 229 |
| *Medicago truncatula* | *MtCCoAOMT9* | Medtr4g085590 | 247 |
| *Medicago truncatula* | *MtCCoAOMT10* | Medtr4g094815 | 248 |
